# A statistical analysis plan for a randomized clinical trial to evaluate the efficacy and safety of ethosuximide in patients with treatment-resistant depression

**DOI:** 10.1097/MD.0000000000016674

**Published:** 2019-08-02

**Authors:** Jiajun Jiang, Zheng Wang, YiYan Dong, Yan Yang, Chee H. Ng, Shuangshuang Ma, Yi Xu, Hailan Hu, Shaohua Hu

**Affiliations:** a Department of Psychiatry, the First Affiliated Hospital, College of Medicine, Zhejiang University; b The Key Laboratory of Mental Disorder's Management of Zhejiang Province; c Brain Research Institute of Zhejiang University; d Center for Neuroscience, Key Laboratory of Medical Neurobiology of the Ministry of Health of China, School of Medicine, Interdisciplinary Institute of Neuroscience and Technology, Qiushi Academy for Advanced Studies; e Mental Health Center, School of Medicine, Zhejiang University, Hangzhou, China; f Department of Psychiatry, University of Melbourne, Melbourne, Victoria, Australia.

**Keywords:** ethosuximide, randomized controlled trial, statistical analysis plan, treatment-resistant depression

## Abstract

**Background and objective:**

A recent striking advance in the treatment of depression has been the finding of rapid antidepressant effects in over 70% of patients with treatment-resistant depression (TRD) using ketamine. However, the potential risk of addiction may limit its clinical use. Recent research revealed that blockade of N-methyl-D-aspartate receptor (NMDAR) dependent bursting activity in the lateral habenula (LHb) could mediate the fast antidepressant effects of ketamine. Further, LHb bursting plays an important role in the pathophysiology of depression that requires both NMDARs and low-voltage-sensitive T-type calcium channels (T-VSCCs). Ethosuximide, which is used to treat absence seizures, is a T-VSCCs inhibitor, may be a novel drug candidate for depression. The objective of this clinical trial is to investigate the efficacy and safety of ethosuximide in patients with TRD.

**Design:**

The study is a single center, randomized, double-blind, placebo-controlled, parallel-group, two-stage clinical trial. Forty patients with TRD will be randomly assigned to Group A (treatment group) or Group B (control group). In the first stage ethosuximide or placebo will be given for 2 weeks. In the second stage, escitalopram (or another antidepressant if escitalopram has been used before) will be given for the next 4 weeks for all trial patients to ensure effective treatment. The primary outcome measure is the Montgomery–Åsberg Depression Rating Scale (MADRS) scores. Secondary outcome measures include the Quick Inventory of Depressive Symptomatology—Self Report score, Hamilton Anxiety Rating Scale scores, individual scores of MADRS, and Young Mania Rating Scale scores. All these scales are measured at baseline and at each treatment visit. Two-way repeated measures analysis of variance is used to analyze the study outcomes.

**Discussion:**

A statistical analysis plan is employed to enhance the transparency of the clinical trial and reduce the risks of outcome reporting bias and data-driven results.

## 1 Introduction

Depression is a highly prevalent mental disorder and is expected to be the second leading cause of disability worldwide by 2020.^[1,2]^ Current conventional antidepressant therapies, based on the monoamine theory, aim to enhance monoaminergic neurotransmission including that of serotonin, noradrenaline (norepinephrine), and dopamine systems.^[3]^ However, there are significant disadvantages in the clinical use of these treatments, including a delay in alleviating depressive symptoms (at least 2–4 weeks), significant adverse effects, and high rates of nonresponse (approximately 30% of patients).^[4]^ In recent years, ketamine has been considered as an important advancement in the last half century because of the rapid antidepressant efficacy (in as little as half an hour) and the efficacy found in over 70% of patients with treatment-resistant depression (TRD).^[5]^ However, ketamine is classified as a psychoactive drug due to its potential addiction,^[6]^ which may limit its application in clinical practice.

H.H.'s research group applied a variety of research neuroscience tools, such as behavioral pharmacology, electrophysiology, and photogenetics, to elucidate the neurobiological mechanism of the rapid antidepressant effect of ketamine. They showed that ketamine quickly improved mood by blocking the N-methyl-D-aspartate receptor (NMDAR) and low-voltage-sensitive T-type calcium channels (T-VSCCs) dependent bursting activity in lateral habenular neurons located in “anti-reward center,” leading to the disinhibition of downstream monoaminergic reward centers. At the same time, animal experiments also confirmed that increased lateral habenula (LHb) burst firing mediated the development of depression. Further research revealed that NMDARs and T-VSCCs worked synergistically to mediate LHb burst firing. Electrophysiological studies using the in vitro brain slice preparation have shown that mibefradil (10 μm), a T-VSCCs inhibitor, could significantly inhibit both the spontaneous bursts and the bursting activity induced by hyperpolarization of LHb neuron. In other studies, mibefradil (10 nmol, 1 μL) injected bilaterally into the lateral LHb to inhibit T-VSCCs-mediated burst firing, resulted in antidepressant efficacy.^[7–9]^ This series of studies provides a framework for developing new rapid-acting antidepressant drugs: local blockade of T-VSCCs in the LHb.

Ethosuximide is a T-VSCCs inhibitor, of which the half maximal inhibitory concentration (IC50) suppressing T-VSCCs current is 0.3 to 1 mM, and the IC50 suppressing sodium channel current on squid axon is 60 mM.^[10]^ Therefore, it has higher selectivity to T-VSCCs. Ethosuximide can enter cerebrospinal fluid through the blood–brain barrier to inhibit T-VSCCs on the LHb neurons, and inhibit the burst firing of the neurons, which can lead to rapid antidepressant efficacy.^[7,10]^ The antidepressant efficacy of ethosuximide has been confirmed in animal experiments, such as chronic restraint stress-induced depression mice^[7]^ and epileptic seizures model animals with depressive symptoms (WAG/Rij rats).^[11]^

In summary, ethosuximide can inhibit the T-VSCCs on the LHb neurons and inhibit the burst activity of neurons, resulting in a rapid antidepressant efficacy. The purpose of this study was to evaluate the clinical efficacy and safety of ethosuximide in patients with TRD.

## 2 Methods

### 2.1 Setting

This is a randomized, double-blind, placebo-controlled, parallel-group, two-stage study conducted by a single center. The study protocol was approved by the Research Ethics Committee of the affiliated Hospital, College of Medicine, Zhejiang University (reference number: 2019/716). The trial which is registered at ClinicalTrials.gov, NCT03887624 aims to investigate the efficacy and safety of ethosuximide compared to placebo in patients with TRD, and it will be conducted in China. The clinic trial was supported by the grants of the National Key Basic Research Program (2016YFC1307100), the Key Research Project of Zhejiang Province (2015C03040), and the National Clinical Research Center for Mental Health Disorders (2015BAI13B02).

### 2.2 Study population

**Figure 1:**
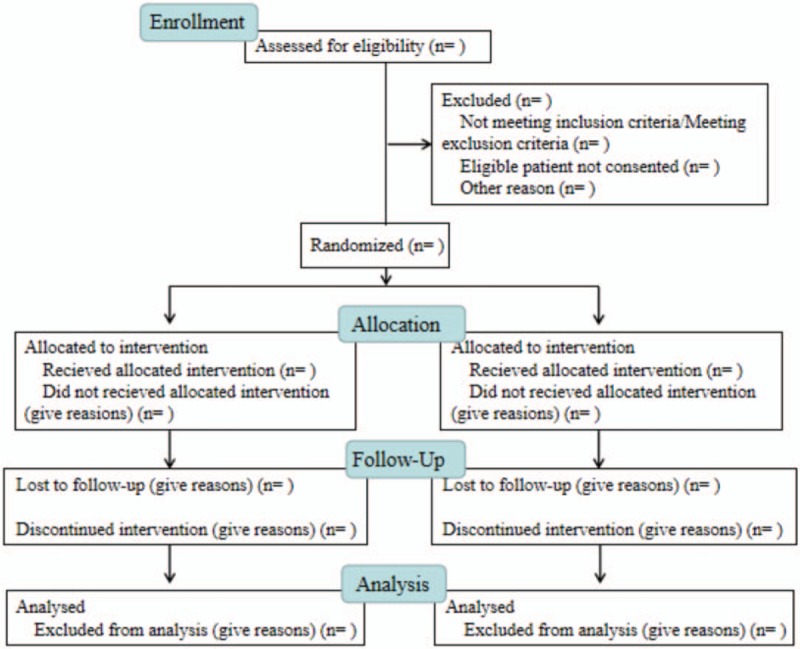
CONSORT flow chart of trial participants. CONSORT = Consolidation Standard of Reporting Trials.

Participants who meet the eligibility criteria and sign the informed consent will be enrolled in the trial. Eligibility criteria including inclusion criteria, exclusion criteria, withdrawal criteria, and suspension criteria have been listed in the study protocol.^[12]^ The flow of participants is illustrated in a flow diagram according to the Consolidation Standard of Reporting Trials (CONSORT) (Fig. 1).

### 2.3 Interventions

Forty patients with TRD will be recruited and randomly assigned to Group A (treatment group) or B (control group) at a ratio of 1:1 by Statistical Analysis System (SAS) software (Hangzhou, China). In the study, ethosuximide or placebo will be given for 2 weeks in Group A or B, respectively, and escitalopram (or another antidepressant if escitalopram has been used before) will be given for the next 4 weeks in each group.

Group A: In the first stage, ethosuximide will be taken orally twice daily. The plan is to start the dosage of the drug at 500 mg on the morning of day 1, then the dosage will be increased to 750 mg at night. On day 2 and 3 the dosage will be 750 mg twice daily. On day 4, the dosage will be 750 mg in the morning and 1000 mg at night. From day 5 to day 14 the dosage will remain at 1000 mg twice daily. In the second stage, participants will take escitalopram in the morning for the next 4 weeks. The dosage of escitalopram will start at 10 mg/day and then increased to 20 mg/day by day 4 without interruption.

Group B: In the first stage, the placebo medications are given in the same way as ethosuximide. In the second stage, participants will take escitalopram in the morning for the next 4 weeks. The dosage of escitalopram will start at 10 mg/day and then increased to 20 mg/day by day 4.

**Figure 2:**
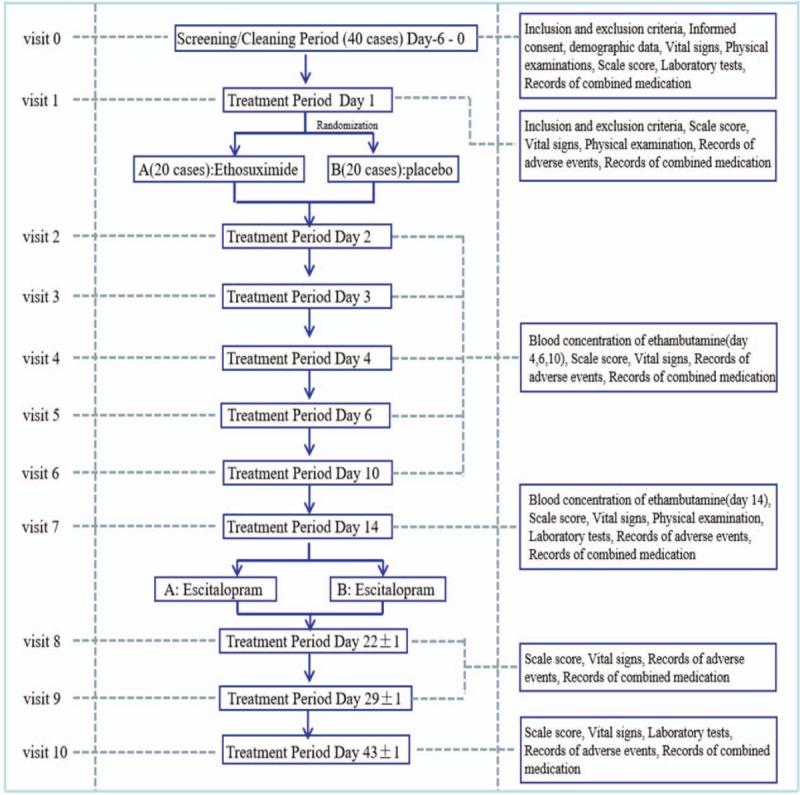
A detailed description of the experiment process.

Please see the flow chart of the trial (Fig. 2) for a detailed description of the experiment process.

### 2.4 Data collection

Demographics and baseline characteristics of participants to be collected are listed in Table 1. Primary outcome will be the change in the Montgomery–Åsberg Depression Rating Scale (MADRS) scores at baseline compared to each treatment time point, while secondary outcomes will be the changes in the Quick Inventory of Depressive Symptomatology—Self Report, Hamilton Anxiety Rating Scale, individual MADRS items and Young Mania Rating Scale scores at baseline compared to each treatment time point.

**Table 1:**
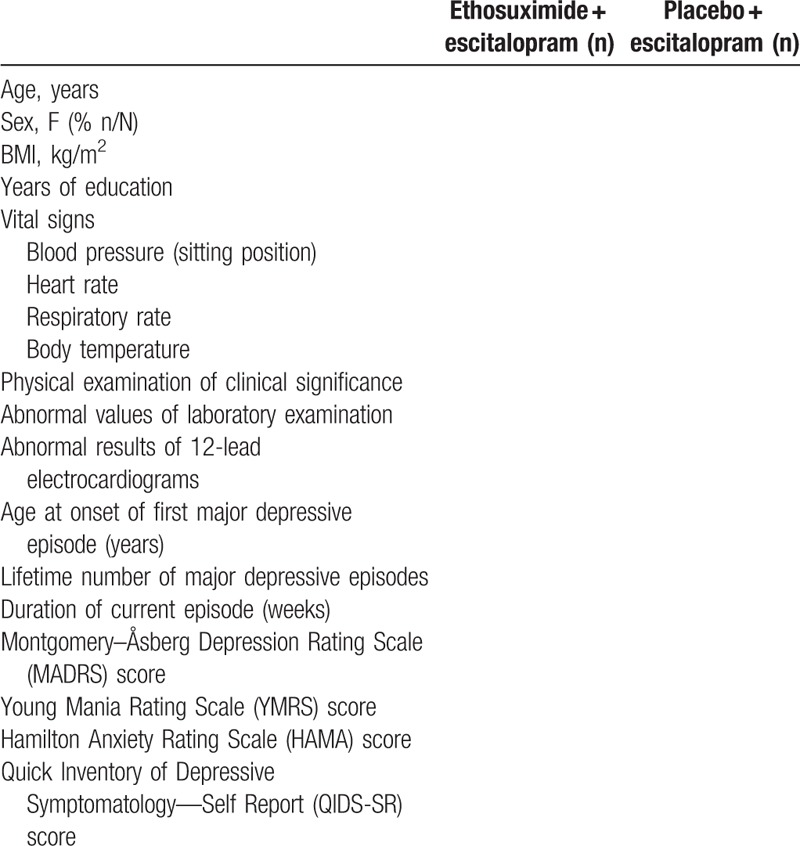
Baseline characteristics.

Vital signs, physical examination, laboratory tests, and 12-lead electrocardiograms will be conducted on all participants. Adverse events occurred in Group A and B will be summarized according to following contents:

1. any treatment emergent adverse events,
2. related treatment emergent adverse events,
3. serious treatment emergent adverse events,
4. adverse events leading to treatment discontinuation,
5. adverse events leading to death,
6. any treatment emergent adverse events by severity, and
7. related treatment emergent adverse events by severity.

The principles for dealing with missing data are based on the practical guide described by Jakobsen et al.^[13]^

### 2.5 Statistical analysis plan

The analysis will be performed after data collection is completed. The latest version of SPSS statistics will be used for data analysis, and all data analysts are blinded to treatment allocation. All analysis results will be two-tailed, with a significance level at 0.05 and 95% confidence intervals.

The research data will be analyzed on the basis of intention-to-treat (ITT) principle. The ITT population will include all randomized participants who received at least 1 scale score or 1 intervention of the study medication. The per-protocol (PP) analysis is another analysis method. Participants who do not receive treatment or do not provide any data of the trial will be excluded from the PP population. Safety data will be collected from participants who received at least 1 scale score or 1 intervention of the study medication, and analyzed at each time point.

The efficacy of the study drugs will be evaluated by analyzing the changes of scales scores from baseline to each treatment point. Two-way repeated measures analysis of variance (ANOVA) will be used to compare the efficacy between experimental and control group; intervention measures will be defined as inter-group variables, and time points of scale evaluation as inner-group variables. First, the scale scores of each participant at baseline and each treatment point be listed (Table 2), and the average value of each group will be calculated. Then, values of sum of squares of deviation from mean, degree of freedom, and mean square will be calculated. After calculating F values of inter-group factors or inner-group variables, the corresponding *P* values by querying F value threshold table and statistical inferences will be determined. It should be noted that the degree of freedom of inner-group variables needs to be corrected in the statistical test.

**Table 2:**
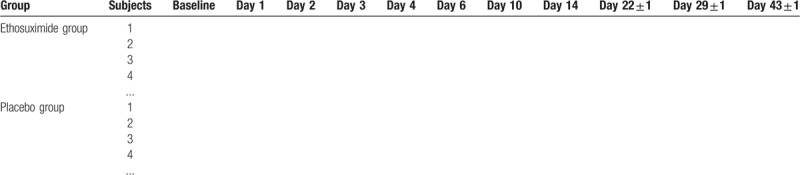
The scale scores of each participant at baseline and each treatment point.

## 3 Discussion

The objective of this randomized clinical trial is to evaluate the efficacy and safety of ethosuximide in patients with treatment-resistant depression. The statistical analysis plan (SAP) completed before the initiation of the trial is employed to enhance the transparency of the clinical trial, and avoid risks of reporting bias. We will analyze the data of this clinical trial according to the protocol reported in this article. This detailed statistical plan includes 1 minor change to the statistical analysis procedures as described briefly in the trial register and the published protocol. The register indicates that independent-sample *t*-test will be used to evaluate the efficacy of ethosuximide, but instead two-way repeated measures ANOVA will be used. The SAP registered in ClinicalTrials.gov will be updated to ensure consistency.

## Author contributions

**Conceptualization:** YiYan Dong, Yan Yang, Shuangshuang Ma.

**Supervision:** Hailan Hu, Shaohua Hu.

**Writing – original draft:** Jiajun Jiang.

**Writing – review & editing:** Zheng Wang, Chee H. Ng, Shaohua Hu.
